# Improvement of medical students' performance in simulated patient interviews by pre-clinical communication training

**DOI:** 10.5116/ijme.6299.c15f

**Published:** 2022-06-17

**Authors:** Pedro Brotons, Montserrat Virumbrales, Marta Elorduy, Sandra Díaz de Castellví, Pau Mezquita, Emili Gené, Albert Balaguer

**Affiliations:** 1 Department of Medicine, School of Medicine and Health Sciences, Universitat Internacional de Catalunya, Barcelona, Spain

**Keywords:** Medical students' performance, simulated patient interviews, pre-clinical communication training

## Abstract

**Objectives:**

To compare the communication skills shown by medical students during simulated patient interviews between those who received training in communication during the preclinical years and those who did not.

**Methods:**

A retrospective study was conducted to analyze the communication skills of several cohorts of fourth-year medical students from Universitat Internacional de Catalunya during simulated patient interviews. Out of a total of 477 students included in the study, 229 (48%) had received training in communication skills through a 60-hour elective course during the preclinical second year, while the remaining 248 (52%) had received none. Communication skills were assessed by an evaluation team using a numerical scale (0 to 10) that included eight categories: "verbal", "non-verbal", "empathy", "concreteness", "warmth", "message content", "assertiveness", and "respect". Scores obtained by trained and non-trained students were compared using the t-test.

**Results:**

A trend towards obtaining better results was observed among students who had received communication training (mean score: 6.98/10) versus none (6.83/10, t_(1,869)_=-1.95, p=0.05). Non-trained male students obtained significantly lower mean scores than non-trained females in the categories of "respect" (7.48/10 vs. 7.83/10, t_(968)_=-2.89, p<0.01), "verbal communication" (6.87/10 vs. 7.15/10, t_(968)_=-2.61, p=0.01), “warmth” (6.53/10 vs. 6.95/10, t_(968)_=-3.40, p<0.01), and "non-verbal communication" (6.49/10 vs. 6.79/10, t_(968)_=-2.48, p=0.01). Trained female and male students had similar scores.

**Conclusions:**

Training in communication skills during the preclinical years may improve fourth-year students' performance in simulated interviews with patients, particularly among males. These results demonstrate the importance of introducing specific training in communication skills early in the undergraduate medical curriculum.

## Introduction

Effective communication between physician and patient is important to establish an adequate diagnosis,^1^ facilitate therapeutic decision making,^2^ ensure patient satisfaction and adherence to treatment,^3^^,^^4^ and provide healthcare with quality and effectiveness.^5^ The clinical interview is the main physician-patient encounter that allows for building mutual trust, collecting and transmitting information, and explaining and planning treatment.^6^^,^^7^The physician's ability to communicate effectively with the patient during the clinical interview is closely associated with a display of other interpersonal competencies such as respect and empathy.^8^^,^^9^

Learning communication skills has become an essential component in the curricula of many medical schools across different countries in recent decades.^10^^,^^11^   In recent years, the simulation of the clinical interview has proved to be a very useful methodology to train medical students in communication skills,^12^^,^^13^  particularly when it is complemented with teacher feedback and student self-reflection on the lived experience.^8^^,^^9^However, courses and activities for the development of communication skills are often implemented in the last years of the undergraduate medical curriculum as a preparation or in parallel to clinical clerkships. There is a need to investigate the potential of early training in communication skills to improve students' performance before they become exposed to simulated or real-life clinical situations.

The objective of this study was to compare the communication skills shown by medical students during simulated patient interviews between those who received training in communication during the preclinical years and those who did not.

## Methods

### Study design

A retrospective longitudinal study was conducted to analyze the communication performance of several cohorts of fourth-year medical students during simulated patient interviews between 2015 and 2020. All students were followed back and assigned to either a group trained in communication skills in the second year or a non-trained group.

### Study setting

The study site was the School of Medicine and Health Sciences of Universitat Internacional de Catalunya (UIC). This institution, located in Barcelona (Spain), has set as one of the central missions of its degree in Medicine to train students so that they establish a physician-patient relationship based on empathy, compassion and a vocation for service, with a holistic vision of the person. The medical school curriculum at UIC lasts six years, including a two-year preclinical stage and clerkships from the third to the sixth year. During the preclinical stage, a 60-hour elective course named "Communication in clinical practice" is offered in the second year to sensitize students about the importance of communication in the relationship with patients and their families. Likewise, during the clinical stage, a required course named "Family and Community Medicine" is delivered in the fourth year to provide students with an opportunity to become familiar with different types of communication strategies in the context of primary care, including the conduction of simulated patient interviews.

### Study participants and sample size

A total of 477 fourth-year students participated in the simulated clinical interview activity, including 229 (48.0%) that had received prior communication training through the second-year elective course and 229 (48.0%) who had not. Gender distribution was similar in the group that had been previously trained (women, n = 146, 48.2%; men, n = 83, 47.7%) and the non-trained group (women, n = 151, 51.8%; men, n = 146, 48.2%), and did not vary significantly across academic years. Details of study participants are shown in Table 1. No sampling procedure was implemented since all eligible students were recruited for the study. Overall, 1861 scores of students' performance were considered for analysis.

All the identifying information of the students was duly anonymized. Ethical approval was obtained from the Institutional Review Board of the UIC School of Medicine and Health Sciences.

**Table 1:** Characteristics of students

Characteristic	No.	Trained Group No. (%)	Untrained Group No. (%)
Gender	477	229 (48.0)	248 (52.0)
Male	174	83 (47.7)	91 (52.3)
Female	303	146 (48.2)	157 (51.8)
Academic year	477	229 (48.0)	248 (52.0)
2018-19	97	52 (53.6)	45 (46.4)
2017-18	93	48 (51.6)	45 (48.4)
2016-17	97	43 (44.3)	54 (55.7)
2015-16	101	45 (44.6)	56 (55.5)
2014-15	89	41 (46.1)	48 (53.9)

Simulated patient interview activity

The simulated clinical interview activity was organized as a succession of four scenarios for primary care encounters of students with chronic, acute, functional, and difficult patients.^14^ For each scenario, four different clinical cases were designed in order to avoid the exchange of information between the students. All clinical cases could be handled in a primary care setting without the need to refer the patient to hospital care. The activity was developed over several sessions in which the students were divided into four groups of 16 students. Each student had four 10-minute clinical interviews, one with each type of simulated patient. The rounds of interviews simultaneously involved four students, one in each specific scenario, while the rest of the group (12 students) waited in a classroom next door. Through these interviews the simulated patients assessed the communication skills of all the students, those who took the elective course and those who did not. The simulated patients were blinded to the prior status of the students in terms of communication training. All simulated patients were healthcare professionals with previous simulation and role-playing training and training in the interpretation and evaluation of clinical scenarios.

### Outcome and exposure variables

The outcome of the study was the assessment of students' communication performance during the interviews. The performance of students was measured by simulated patients using a numerical scale from 0 to 10 (where 0 is the lowest score and 10 is the highest). The scale was specifically designed for this study and included eight categories of communication: "verbal", "non-verbal", "empathy", "concreteness", "warmth", "message content", "assertiveness", and "respect". Scores from all categories were averaged into a global "communication" score. Table 2 describes the varied categories of communication that were scored.

**Table 2:** Description of the categories built into Global Communication

Category	Description
Verbal communication	The student addresses the patient using the proper tone, pitch, volume, speed and pausing and avoids the use of unnecessary or unfamiliar technical terms. The patient altogether feels comfortable with the way information is provided to him or her.
Non-verbal communication	The student uses the appropriate body language, which includes eye contact, facial expressions, hand gestures, distance and body position with respect to the patient, in accordance with the situation of the interview and the setting where it takes place.
Empathy	The student understands the patient's response to a situation and responds to their emotions appropriately.
Concreteness	Both the student and the patient are specific and definite in communicating, rather than vague and general. A mutual exchange of information based on the facts fulfils the objectives of the clinical interview on both sides.
Warmth	The student shows friendliness and kindness when dealing with the patient, making him or her feel comfortable during the interview.
Message content	The student shows competence in the subject of the interview and is able to apply his or her technical knowledge to the patient's best interest.
Assertiveness	The student is able to express his or her own thoughts and is willing to stand up for his or her own interests and those of others in a firm and civil manner. The student is aware of the rights of the patient and is willing to work on resolving conflicts, particularly when confronted with patients exhibiting unjustified demands, threats of the lawsuit or false accusations.
Respect	The student is honest in providing care and advice to the patient and accepts the patient's thoughts and feelings without judgment or punishment.

The exposure variables were the previous training status of students in communication skills during the second year or none, students' gender, and the academic year in which they took the simulated clinical interview activity.

### Data collection and analysis

Simulated patients evaluated the students during the 5-minute break between interviews and posted the scores and the students' identification data online on a Google Forms questionnaire. Data of exposure variables were retrieved from the academic records of the study site.

Students' performance scores (global and for each dimension) are described as mean values and standard deviations. Students' gender and academic year are described as proportions. The association between students' communication scores (global and for each specific dimension) and preclinical training in communication skills or none was assessed using the Student's T-test after confirming equality of variances by the Levene's test. A sub-analysis by gender was also performed to identify possible differences in scoring between male and female students. Data analysis was carried out, establishing a level of significance of p<0.05 and using the statistical package Stata v. 15.

## Results

Overall, a trend towards obtaining significantly better results was observed for the aggregated category of "communication" among the trained students (mean score: 6.98/10) compared with those non-trained (6.83/10, t_(1869)_=-1.95, p=0.05). Differences in mean score between the two groups were significant for "message content" (trained group: 6.86/10; non-trained: 6.69/10, t_(1,860)_=-2.26, p =0.02) but minor for the rest of the dimensions.

The categories of communication that obtained the best average scores among students who had been previously trained in communication skills in the second year were "respect" (7.85/10, SD=1.55) and "verbal communication" (7.16/10, SD=1.46). Both categories also best scored among students who had not been trained ("respect": 7.71/10, SD=1.72; "verbal communication": 7.05/10, SD=1.53). In contrast, the category of "empathy" showed the lowest mean scores, both in trained (6.67/10, SD=1.96) and non-trained students (6.53/10, SD=2.01).

Among the students that had not been previously trained in communication skills in the second year, females obtained significantly higher mean scores than males in the categories of "respect" (7.83/10 vs. 7.48/10, t_(968)_=-2.89, p < 0.01), "verbal communication" (7.15/10 vs. 6.87/10, t_(968)_=-2.61, p = 0.01), “warmth” (6.95/10 vs. 6.53/10, t_(968)_=-3.40. p < 0.01), and "non-verbal communication" (6.79/10 vs. 6.49/10, t_(968)_=-2.48, p = 0.01). Mean scores were similar for all communication categories in trained females and males. Scores of students according to preclinical communication training and sex are shown in Table 3.

The lowest mean scores of the students for any of the eight communication categories according to the type of patient were always recorded in the interviews with simulated patients that were difficult to deal with. "Empathy" was again the communication category in which the lowest mean score was observed in interviews with difficult-to-deal-with patients and functional patients. The worst valued category in the interviews with acute patients was "concreteness", while "non-verbal communication" and "assertiveness" had the lowest scores in scenarios with chronic patients.

**Table 3:** Students' performance in simulated patient interviews according to preclinical communication training and sex

Preclinical communication training	All trained	None trained	p value	diff (95% CI)	Trained females	Trained males	p value		Untrained females	Untrained males	p value	diff (95% CI)
	Mean score (SD)	Mean score (SD)			Mean score (SD)	Mean score (SD)		diff (95% CI)	Mean score (SD)	Mean score (SD)		
Communication	6.98 (1.58)	6.83 (1.65)	0.05	0.15 (0.00,0.29)	7.05 (1.47)	6.86 (1.75)	0.11	0.19 (-0.04,0.41)	6.92 (1.58)	6.68 (1.76)	0.03	0.24 (0.03,0.47)
Verbal	7.16 (1.46)	7.05 (1.53)	0.11	0.11 (-0.03,0.25)	7.19 (1.42)	7.09 (1.54)	0.35	0.10 (-0.11, 0.30)	7.15 (1.44)	6.87 (1.68)	0.01	0.28 (0.07,0.49)
Nonverbal	6.79 (1.68)	6.68 (1.76)	0.18	0.11 (-0.05,0.26)	6.87 (1.64)	6.64 (1.75)	0.05	0.23 (0.00,0.47)	6.79 (1.65)	6.49 (1.92)	0.01	0.30 (0.06,0.54)
Empathy	6.67 (1.96)	6.53 (2.01)	0.12	0.14 (-0.04,0.32)	6.75 (1.85)	6.51 (2.13)	0.09	0.24 (0.04,0.52)	6.62 (1.94)	6.35 (2.12)	0.05	0.27 (0.00, 0.54)
Concreteness	6.90 (1.73)	6.75 (1.76)	0.07	0.15 (-0.01,0.31)	6.89 (1.69)	6.92 (1.81)	0.82	-0.03 (-0.27,0.21)	6.80 (1.70)	6.66 (1.86)	0.27	0.14 (-0.10, 0.37)
Warmth	6.95 (1.68)	6.80 (1.79)	0.06	0.15 (-0.01,0.31)	7.02 (1.61)	6.82 (1.80)	0.11	0.20 (0.04,0.43)	6.95 (1.73)	6.53 (1.88)	<0.01	0.42 (0.18, 0.66)
Message content	6.86 (1.67)	6.69 (1.72)	0.02	0.17 (0.02,0.33)	6.89 (1.63)	6.82 (1.73)	0.56	0.07 (-0.16,0.30)	6.77 (1.68)	6.54 (1.78)	0.06	0.23 (0.00, 0.45)
Assertiveness	6.92 (1.66)	6.76 (1.71)	0.05	0.16 (0.00,0.31)	6.94 (1.61)	6.87 (1.75)	0.54	0.07 (-0.16,0.31)	6.84 (1.63)	6.62 (1.83)	0.06	0.22 (0.00, 0.45)
Respect	7.85 (1.55)	7.71 (1.72)	0.06	0.14 (0.00,0.29)	7.92 (1.44)	7.74 (1.73)	0.11	0.18 (-0.04,0.40)	7.83 (1.59)	7.48 (1.91)	<0.01	0.35 (0.11, 0.58)

## Discussion

The present study suggests that medical students who receive training in communication skills in the preclinical years may gain higher communication competence to deal with patients in the clinical years. Early communication training could especially benefit male students, who presented lower communication abilities at baseline in our study but performed similarly as females once they had been trained. "Empathy", as the patient's perception of being understood by the student, was revealed to be a crucial category with significant potential for improvement in both students that showed good and limited performance in communication.

Our results are in partial agreement with those of other similar studies that evaluated the communication abilities of medical students. In a clinical trial on reporting bad news carried out in the United States, students in the clinical rotation who had previously been trained in communication skills received significantly higher ratings from standardized patients than their control counterparts.^15^ In the same country, the competence of fourth-year students trained in the communication of deaths was significantly better valued than that of the untrained.^16^ In contrast, a clinical trial with third-year students who underwent a short one-hour communication workshop found no difference in their subsequent competence to communicate with standardized patients who simulated unwanted pregnancies compared with students who had no prior training.^17^ These results indicate that it is important to introduce communication training activities early in the medical curriculum and assign them the required credit hours to be effective.

The scores collected among students seem to indicate that women developed better communication skills than their male colleagues, especially in the absence of previous training in these skills. In turn, results showed that the communication abilities of the students previously sensitized about the importance of  communication  were similar regardless of

their gender. Higher communication competence shown by women during undergraduate medical training could be an early indication of the capability of female physicians to communicate more openly and empathetically with their patients than their male counterparts, as previously reported in a systematic review.^18^ Likewise, females predominated among the students who chose to take second-year communication training in our study, which suggests their comparatively higher interest to improve in this specific interpersonal skill. This result is in agreement with the literature since several studies have described an attitude of greater sensitivity and interest of female medical students in the development of communication skills.^19^^-^^21^

The results revealed that the students found particular problems in communicating with patients challenging to deal with them. Other studies have analyzed training activities to teach students to cope with difficult situations, such as the communication of bad news. They have tested the effectiveness of such training activities with varied outcomes.^15^^,^^16^^,^^22^ It should be noted that our students also showed limited empathy in the interviews with difficult-to-deal-with patients, as well as with functional patients. The close relationship between empathy and effective communication has been previously described in studies with students and health professionals, stressing the importance of this dimension to improve patient satisfaction and acceptance of bad prognoses.^3^^,^^23^^-^^25^ Therefore, sensitization on empathy may be an essential aspect to be considered when designing and including courses and activities to improve the communication skills of medical students.

This study presents a number of strengths and limitations. The longitudinal perspective of the design and the extensive size of the study population are significant strengths that underscore the internal validity of the results. The authenticity of the interview conducted with simulated patients, who were health professionals specifically trained in simulation, interpretation, and evaluation of clinical scenarios, is also considered a strength. These simulated patients had the ability to interact dynamically with the students based on the responses that were given to their demands, providing a high degree of realism to the interviews. To be noted, blinding simulated patients to students' assignment to the group previously trained in communication skills or to the untrained group also minimized the risk of information bias. A first limitation of the study is the use of an instrument designed to measure students' communication performance in simulated interviews for this specific study. Although this instrument was not previously validated, its design was based on the consideration of a number of categories that are generally accepted in the literature to be essential for communication assessment. A second limitation could be that we were not able to dissociate the effect of communication training in the second year from other exposures that students could have experienced in the timeframe between the second and the fourth academic year. In the third place, it must be considered that the monocentric nature of the study could limit the generalization of results.

## Conclusions

Training medical students, especially males, in communication skills during the preclinical years may improve their communication competence when they interact with patients later on in the clinical years of the curriculum. Limited capability to empathize could undermine students' global competence for effective communication. The results of this study demonstrate the importance of training in communication from the first years of the undergraduate medical curriculum. New studies are encouraged to analyze whether this beneficial effect is maintained over time and in real-life communication with patients and their families during the clinical clerkships.

### Conflict of Interest

The authors declare that they have no conflict of interest.

## References

[1] Peterson MC, Holbrook JH, Von Hales D, Smith NL, Staker LV (1992). Contributions of the history, physical examination, and laboratory investigation in making medical diagnoses.. West J Med.

[2] National Health Service. 2012. Liberating the NHS: no decision about me, without me - further consultation on proposals to secure shared decision-making. [Cited 16 December 2021]; Available from: https://assets.publishing.service.gov.uk/government/uploads/system/uploads/attachment_data/file/216980/Liberating-the-NHS-No-decision-about-me-without-me-Government-response.pdf.

[3] Pollak KI, Alexander SC, Tulsky JA, Lyna P, Coffman CJ, Dolor RJ, Gulbrandsen P, Ostbye T (2011). Physician empathy and listening: associations with patient satisfaction and autonomy.. J Am Board Fam Med.

[4] Street RL, Makoul G, Arora NK, Epstein RM (2009). How does communication heal? Pathways linking clinician-patient communication to health outcomes.. Patient Educ Couns.

[5] Richardson WC, Berwick DM, Bisgard JC, Bristow L, Buck CR, Cassel CK, et al. Crossing the quality chasm: a new health system for the 21st Century. Washing-ton DC: National Academies Press; 2001. 25057539

[6] Kurtz S, Silverman J, Draper J. Teaching and learning communication skills in medicine. Abingdon, UK: Radcliffe Medical Press; 1998.

[7] Makoul G. Communication research in medical education. In: Jackson L, DuGy BK, editors(s). Health communication research: a guide to developments and directions. Westport, CT: Greenwood Press; 1998.

[8] Kurtz S, Silverman J, Benson J, Draper J (2003). Marrying content and process in clinical method teaching: enhancing the Calgary-Cambridge guides.. Acad Med.

[9] Makoul G (2001). Essential elements of communication in medical encounters: the Kalamazoo consensus statement.. Acad Med.

[10] Laidlaw A, Hart J (2011). Communication skills: an essential component of medical curricula. Part I: Assessment of clinical communication: AMEE Guide No. 51.. Med Teach.

[11] Borrell-Carrió F, Clèries X, Paredes-Zapata D, Borrás-Andrés JM, Sans-Corrales M, Mascort-Roca J.J. Bologna process (VI): learning communication for health in the degree of Medicine [Proceso de Bolonia (VI): aprendiendo comunicación para la salud en el Grado de Medicina]. Educ Med. 2012;15:197-201.

[12] Ryan CA, Walshe N, Gaffney R, Shanks A, Burgoyne L, Wiskin CM (2010). Using standardized patients to assess communication skills in medical and nursing students.. BMC Med Educ.

[13] Morrow JB, Dobbie AE, Jenkins C, Long R, Mihalic A, Wagner J (2009). First-year medical students can demonstrate EHR-specific communication skills: a control-group study.. Fam Med.

[14] Gené E, Olmedo L, Pascual M, Azagra R, Elorduy M, Virumbrales M (2018). [Evaluation of clinical communication skills in medical students with simulated patients].. Rev Med Chil.

[15] Colletti L, Gruppen L, Barclay M, Stern D (2001). Teaching students to break bad news.. Am J Surg.

[16] Hobgood CD, Tamayo-Sarver JH, Hollar DW, Sawning S (2009). Griev_Ing: death notification skills and applications for fourth-year medical students.. Teach Learn Med.

[17] Shaddeau A, Nimz A, Sheeder J, Tocce K (2015). Effect of novel patient interaction on students' performance of pregnancy options counseling.. Med Educ Online.

[18] Roter DL, Hall JA, Aoki Y (2002). Physician gender effects in medical communication: a meta-analytic review.. JAMA.

[19] Rees C, Sheard C (2002). The relationship between medical students' attitudes towards communication skills learning and their demographic and education-related characteristics.. Med Educ.

[20] Cleland J, Foster K, Moffat M (2005). Undergraduate students' attitudes to communication skills learning differ depending on year of study and gender.. Med Teach.

[21] Ruiz-Moral R, Monge Martin D, Garcia de Leonardo C, Denizon S, Cerro Pérez A, Caballero Martínez F (2021). Medical students' attitudes towards communication skills training: a longitudinal study with one cohort.. GMS J Med Educ.

[22] Gorniewicz J, Floyd M, Krishnan K, Bishop TW, Tudiver F, Lang F (2017). Breaking bad news to patients with cancer: a randomized control trial of a brief communication skills training module incorporating the stories and preferences of actual patients.. Patient Educ Couns.

[23] Bas-Sarmiento P, Fernández-Gutiérrez M, Díaz-Rodríguez M (2019). Teaching empathy to nursing students: A randomised controlled trial.. Nurse Educ Today.

[24] Lippe M, Farya P, Jennifer M, Stanley A, Barbara J, Boone G (2020). Communicating oncologic prognosis with empathy: a pilot study of a novel communication guide.. Am J Hosp Palliat Care.

[25] Nixon J, Gray L, Turner J, Bernard A, Scaife J, Cartmill B (2020). Communicating actively responding empathically (CARE): comparison of communication training workshops for health professionals working in cancer care.. J Cancer Educ.

